# Supplementary material for: Was R < 1 before the English lockdowns? On modelling mechanistic detail, causality and inference about Covid-19
Source: PLoS One. 2021 Sep 22;16(9):e0257455. doi: 10.1371/journal.pone.0257455 (PMC8457481; doi:10.1371/journal.pone.0257455)
Supplement: S1 Appendix — (PDF) [file pone.0257455.s001.pdf]

Supplementary Appendices for
'Was $R < 1$ before the English lockdowns? On modelling mechanistic detail, causality and inference about Covid-19', S.N. Wood & E.C. Wit

A Dynamic model details

This appendix gives full details of the dynamic model structure and computations not covered in the main paper, including the model compartments for care homes, hospitals and testing, as well as the output variables predicting the data.

A.1 Force of infection

Here is the force of infection term for for classes 0 to 16, written out in a form where the sensitivities follow easily.

$$\lambda_i(t) = b(t)\left\{\sum_{j=0}^{16} C_{i,j}I_j + c_i^{\text{chw}}I_{17} + \epsilon C_{i,16}I_{18}\right\}$$

For care home workers, class 17,

$$\lambda_{17}(t) = b(t)\sum_{j=0}^{16} C_{i,j}I_j + m_{\text{chw}}I_{17} + m_{\text{chw}}I_{18}$$

and for care home residents, class 18,

$$\lambda_{18}(t) = \epsilon b(t)\sum_{j=0}^{16} C_{i,16}I_j + m_{\text{chw}}I_{17} + m_{\text{chr}}I_{18}$$

The sensitivities require the derivatives of these terms w.r.t. the parameters $\boldsymbol{\theta}$ (i.e. ϵ, m_{chw}, m_{chw} and the parameters of $b(t)$ - sensitivities to further parameters are obviously zero).

A.2 The contact matrix, C

C is based on the POLYMOD survey (Mossong et al., 2017) accessed through R package `socialmixr` (Funk, 2020), which had 1011 participants in the UK, who each recorded their contacts on one day. There were 7 participants in the 75-80 age group and none over 80. Knock et al. (2020) are vague about exactly how C_{ij} is obtained, but given the statement that it is symmetric there is really only one sane option.

Let A_{ij} denote the average number of contacts of someone in age class i with someone in age class j. Because population sizes may differ between age classes, $A_{ij} \neq A_{ji}$ in general. Let S_i^0 denote the population (initially susceptible) in class i. The total number of contacts between members of class i and class j is $T_{ij} = A_{ij}S_i^0$. Obviously for total contacts $T_{ij} = T_{ji}$. In reality the polymod estimates of T_{ij} and T_{ji} will usually be different because the study population is not closed and there are likely to be some recording errors. It therefore makes sense to replace both by their average.

The number of infections generated in class i by class j is proportional to the total contacts between S_i and I_j. That is the total number of contacts between the classes, multiplied by the proportion that are between S_i and I_j,

$$T_{ij}\frac{S_i}{S_i^0}\frac{I_j}{S_j^0} = C_{ij}S_iI_j$$

by definition of C_{ij}.The contacts for care home workers with the general population are set to the mean of the C_{ij} for the ages 20-65.

In practice there is no data on 80+ with 80+ contacts. The preceding age class has 10% of contacts within age class, so this proportion could be assumed to apply to the 80+ also, and was the assumption made here. Knock et al. (2020) do not document what they did. The `sircovid` package accompanying Knock et al. (2020) has a `carehomes_parameters()` function. Among other things this returns a matrix m, which is *exactly* the $\mathbf{C}$ matrix defined above, up until age 70, but thereafter something undocumented appears to have been done and it is not clear if this was the $\mathbf{C}$ used or not. In sensitivity testing it made negligible difference whether we used the `sircovid` matrix or the version just described.

A.3 Computing R

Knock et al. (2020) do not describe exactly what was done to compute R, but section 4.1 of Diekmann et al. (1990) provides what is required. For the current model we conceptually divide the population into those who will show symptoms and those who will not, so that we have E_A and E_C (exposed eventually asymptomatic and exposed eventually symptomatic). This division does not change the dynamics. Let $\mathbf{S}$ denote the vector of susceptibles in each class. We define the matrix

$$\mathbf{K} = \begin{pmatrix} (1-p_c)\text{diag}(\mathbf{S})\mathbf{C}/\gamma_A & (1-p_c)\text{diag}(\mathbf{S})\mathbf{C}/\gamma_C \\ p_c\text{diag}(\mathbf{S})\mathbf{C}/\gamma_A & p_c\text{diag}(\mathbf{S})\mathbf{C}/\gamma_C \end{pmatrix}.$$

R is the dominant eigenvalue of $\mathbf{K}$. Checking by simulation, the epidemic does indeed decline when $R < 1$ and increase when $R > 1$. Note that except when $R = 1$, anything that lengthens the assumed generation time requires a corresponding increase(decrease) in R in order to achieve the same epidemic growth(shrinkage) rate in time. The generation times assumed by Knock et al. (2020) are fairly long, and get longer after the rate parameter corrections described in the main paper.

To average R from different regions we need to weight by the total number of infectives in each region, since conceptually R is the mean number of new infections caused by each infective.

A.4 Care home dynamics

The first additional compartments relate to care home deaths. The equations are given for each age class, but actually only apply to class $i = 18$, care home residents.

$$\dot{G}_D^{i,1} = p_H^{max}\psi_H^i p_{G_D}^i \gamma_C I_C^i - \gamma_{G_D} G_D^{i,1} \quad (1)$$
$$\dot{G}_D^{i,2} = \gamma_{G_D} G_D^{i,1} - \gamma_{G_D} G_D^{i,2} \quad (2)$$

Because this only applies in a single class there is only one p_{G_D} parameter to estimate. ψ_H^i is given in the Knock et al.supplementary material as `psi_hosp_symp`.

A.5 Hospital dynamics

Now consider the ICU flows ($i = 0 \ldots 17$). Define

$$r(t,\mu) = \begin{cases} 1 & t < t_0 \\ 1 + (1-\mu)(t_0 - t)/(t_1 - t_0) & t_0 \le t \le t_1 \\ \mu & t > t_1 \end{cases}$$

t_0 is set to 1 April and t_1 to 1 June. The reference given to justify this term is a report on a clinical trial that showed modest improvements, but did not finish until July (RECOVERY Collaborative Group, 2020). It is unclear how improvements developed during the trial could have wide-spread consequences before its completion. Despite that caveat, Dennis et al. (2021) do report improvements in mortality rates of hospitalized patients in England over this period, and show that these improvements are not the

result of changes in patient characteristics. However it is difficult to rule out that some component of the change may relate to on the ground changes in the severity of disease required for admission, particularly between peak hospital load and later. Another difficulty is the problem of nosocomial infection (e.g. McKeigue et al., 2021), which became significant over the period of the apparent improvements, but is likely to reduce the apparent IFR, since patients infected in hospital, while vulnerable, are obviously likely to have lower mortality rates than patients admitted to hospital *because* their Covid infection had resulted in serious illness.

In what follows starred states are for cases where Covid-19 has been confirmed by test. The ICU compartments are governed by the following ODEs - here written out in a form such that the sensitivity equations follow by inspection. ICU^i_{pre} is the compartment preceding admission to ICU, $ICU^i_{W_r}$ is the compartment for patients in the ICU who will eventually recover, back on the ward, $ICU^i_{W_D}$ is for ICU patients who will eventually die back on the general ward, ICU_D compartments are for patients who die in the ICU. Parameters μ_{IC}, μ_D, p^{max}_H, p^{max}_{IC}, $p^{max}_{IC_D}$ and $p^{max}_{W_d}$ are free, and we additionally treated $\gamma_{IC_{W_r}}$ as free.

$$\dot{ICU}^i_{pre} = p^{max}_H \psi^i_H (1-p^*) p^{max}_{IC} \psi^i_{IC} r(t,\mu_{IC}) \gamma_C I^i_C - (\gamma_{ICpre} + \gamma_U) ICU^i_{pre} \tag{3}$$

$$\dot{ICU}^i_{pre*} = p^{max}_H \psi^i_H p^* p^{max}_{IC} \psi^i_{IC} r(t,\mu_{IC}) \gamma_C I^i_C - \gamma^{pre}_{IC} ICU^i_{pre*} + \gamma_U ICU^i_{pre} \tag{4}$$

$$\dot{ICU}^i_{W_r} = (1 - p^{max}_{IC_D} \psi^i_{IC_D} r(t,\mu_D))(1 - p^{max}_{W_d} \psi^i_{W_D} r(t,\mu_D)) \gamma^{pre}_{IC} ICU^i_{pre} - (\gamma_{IC_{W_r}} + \gamma_U) ICU^i_{W_r} \tag{5}$$

$$\dot{ICU}^i_{W_r*} = (1 - p^{max}_{IC_D} \psi^i_{IC_D} r(t,\mu_D))(1 - p^{max}_{W_d} \psi^i_{W_D} r(t,\mu_D)) \gamma^{pre}_{IC} ICU^i_{pre*} - \gamma_{IC_{W_r}} ICU^i_{W_r*} + \gamma_U ICU^i_{W_r} \tag{6}$$

$$\dot{ICU}_{W_D} = (1 - p^{max}_{IC_D} \psi^i_{IC_D} r(t,\mu_D)) p^{max}_{W_d} \psi^i_{W_D} r(t,\mu_D) \gamma^{pre}_{IC} ICU^i_{pre} - (\gamma_{IC_{W_D}} + \gamma_U) ICU^i_{W_D} \tag{7}$$

$$\dot{ICU}_{W_{D*}} = (1 - p^{max}_{IC_D} \psi^i_{IC_D} r(t,\mu_D)) p^{max}_{W_d} \psi^i_{W_D} r(t,\mu_D) \gamma^{pre}_{IC} ICU^i_{pre*} - \gamma_{IC_{W_D}} ICU^i_{W_{D*}} + \gamma_U ICU^i_{W_D} \tag{8}$$

$$\dot{ICU}^{i,1}_D = p^{max}_{IC_D} \psi^i_{IC_D} r(t,\mu_D) \gamma^{pre}_{IC} ICU^i_{pre} - (\gamma_{IC_D} + \gamma_U) ICU^{i,1}_D \tag{9}$$

$$\dot{ICU}^{i,2}_D = \gamma_{IC_D} ICU^{i,1}_D - (\gamma_{IC_D} + \gamma_U) ICU^{i,2}_D \tag{10}$$

$$\dot{ICU}^{i,1}_{D*} = p^{max}_{IC_D} \psi^i_{IC_D} r(t,\mu_D) \gamma^{pre}_{IC} ICU^i_{pre*} - \gamma_{IC_D} ICU^{i,1}_{D*} + \gamma_U ICU^{i,1}_D \tag{11}$$

$$\dot{ICU}^{i,2}_{D*} = \gamma_{IC_D} ICU^{i,1}_{D*} - \gamma_{IC_D} ICU^{i,2}_{D*} + \gamma_U ICU^{i,2}_D \tag{12}$$

The next 6 compartments are the step down from ICU to regular ward compartments. They originally involved no free parameters, but we treated γ_{W_r} as free. Subscripts r and D refer to recovery or death.

$$\dot{W}^{i,1}_r = \gamma_{IC_{W_r}} ICU^i_{W_r} - (\gamma_{W_r} + \gamma_U) W^{i,1}_r \tag{13}$$

$$\dot{W}^{i,2}_r = \gamma_{W_r} W^{i,1}_r - (\gamma_{W_r} + \gamma_U) W^{i,2}_r \tag{14}$$

$$\dot{W}^{i,1}_{r*} = \gamma_{IC_{W_r}} ICU^i_{W_r*} - \gamma_{W_r} W^{i,1}_{r*} + \gamma_U W^{i,1}_r \tag{15}$$

$$\dot{W}^{i,2}_{r*} = \gamma_{W_r} W^{i,1}_{r*} - \gamma_{W_r} W^{i,2}_{r*} + \gamma_U W^{i,2}_r \tag{16}$$

$$\dot{W}^i_D = \gamma_{IC_{W_d}} ICU^i_{W_d} - (\gamma_{W_d} + \gamma_U) W^i_D \tag{17}$$

$$\dot{W}^i_{D*} = \gamma_{IC_{W_d}} ICU^i_{W_d*} - \gamma_{W_d} W^i_{D*} + \gamma_U W^i_D \tag{18}$$

Six further compartments are for the hospital general ward, and again depend on free parameters, including new one $p^{max}_{H_d}$. We also treated γ_{H_r} as free. Again subscripts r and D refer to recovery or

death.

$$\dot{H}^i_r = p^{max}_H \psi^i_H (1-p^*)(1-p^{max}_{IC}\psi^i_{IC} r(t,\mu_{IC}))(1-p^{max}_{H_d}\psi^i_{H_d} r(t,\mu_D))\gamma_C I^i_C - (\gamma_{H_r}+\gamma_U)H^i_r \quad (19)$$
$$\dot{H}^i_{r*} = p^{max}_H \psi^i_H p^* (1-p^{max}_{IC}\psi^i_{IC} r(t,\mu_{IC}))(1-p^{max}_{H_d}\psi^i_{H_d} r(t,\mu_D))\gamma_C I^i_C + \gamma_U H^i_r - \gamma_{H_r} H^i_{r*} \quad (20)$$
$$\dot{H}^{i,1}_D = p^{max}_H \psi^i_H (1-p^*)(1-p^{max}_{IC}\psi^i_{IC} r(t,\mu_{IC}))p^{max}_{H_d}\psi^i_{H_d} r(t,\mu_D)\gamma_C I^i_C - (\gamma_{H_D}+\gamma_U)H^{i,1}_D \quad (21)$$
$$\dot{H}^{i,2}_D = \gamma_{H_D} H^{i,1}_D - (\gamma_{H_D}+\gamma_U)H^{i,2}_D \quad (22)$$
$$\dot{H}^{i,1}_{D*} = p^{max}_H \psi^i_H p^* (1-p^{max}_{IC}\psi^i_{IC} r(t,\mu_{IC}))p^{max}_{H_d}\psi^i_{H_d} r(t,\mu_D)\gamma_C I^i_C + \gamma_U H^{i,1}_D - \gamma_{H_d} H^{i,1}_{D*} \quad (23)$$
$$\dot{H}^{i,2}_{D*} = \gamma_{H_d} H^{i,1}_{D*} - \gamma_{H_d} H^{i,2}_{D*} + \gamma_U H^{i,2}_D \quad (24)$$

The recovered compartment is governed by

$$\dot{R}^i = \gamma_A I^i_A + (1-p^{max}_H\psi^i_H)\gamma_C I^i_C + \gamma_{H_r}(H^i_r + H^i_{r*}) + \gamma_{W_r}(W^{i,2}_r + W^{i,2}_{r*}) \quad (25)$$

(this is corrected from Knock et al., 2020).

A.6 Testing compartments

Finally there are compartments used to determine the proportions testing positive in randomized testing. They have no free parameters (S subscript for antibodies, P for PCR).

$$\dot{T}^i_S = \gamma_E E^{i,2} - \gamma_S T^i_S \quad (26)$$
$$\dot{T}_{S+} = p_{S+}\gamma_S T^i_S \quad (27)$$
$$\dot{T}_{S-} = (1-p_{S+})\gamma_S T^i_S \quad (28)$$
$$\dot{T}^i_P = \lambda_i S^i - \gamma_P T^i_P \quad (29)$$
$$\dot{T}^i_{P+} = \gamma_P T^i_P - \gamma_{P+} T^i_{P+} \quad (30)$$
$$\dot{T}^i_{P-} = \gamma_{P+} T^i_{P+} \quad (31)$$

(corrected from Knock et al., 2020).

A.7 Model outputs

The outputs required from all this are as follows, again written in a form in which the sensitivities are immediate. Firstly the rate of new Covid cases in hospital, which is the sum of the flows into the H_{r*}, H_{D*} and ICU_{pre*} states plus all the rate γ_U flows.

$$X_{adm} = \sum_i p^{max}_H \psi^i_H p^* \gamma_C I^i_C + \gamma_U (ICU^i_{pre} + ICU^i_{W_r} + ICU^i_{W_D} + ICU^{i,1}_D + ICU^{i,2}_D + W^{i,1}_r + W^{i,2}_r + W^i_D + H^i_r + H^{i,1}_D + H^{i,2}_D) \quad (32)$$

Note that Knock et al. (2020) modify some of the γ_U plumbing between the ODE statement of the model and its stochastic discretisation, but this does not change the flow totals.

The hospital regular bed occupancy is given by (correcting report notation mutation)

$$X_{hos} = \sum_i H^i_{r*} + H^{i,1}_{D*} + H^{i,2}_{D*} + ICU^i_{pre*} + W^i_{D*} + W^{i,1}_{r*} + W^{i,1}_{r*} \quad (33)$$

But a correctly specified likelihood actually requires regular bed arrivals

$$\sum_i p^{max}_H \psi^i_H p^* \gamma_C I^i_C + \gamma_U ICU^i_{pre} + \gamma_{IC_{W_r}} ICU^i_{W_r*} + \gamma_U W^{i,1}_r + \gamma_U W^{i,2}_r + \gamma_{IC_{W_d}} ICU^i_{W_d*} + \gamma_U W^i_D + \gamma_U H^i_r + +\gamma_U H^{i,1}_D + \gamma_U H^{i,2}_D \quad (34)$$

and departures

$$\sum_i \gamma_{IC}^{pre} ICU^i_{pre*} + \gamma_{W_r} W^{i,2}_{r*} + \gamma_{W_d} W^i_{D*} + \gamma_{H_r} H^i_{r*} + \gamma_{H_d} H^{i,2}_{D*} \tag{35}$$

ICU occupancy is

$$X_{icu} = \sum_i ICU^i_{W_{r*}} + ICU^i_{W_{D*}} + ICU^{i,1}_{D*} + ICU^{i,2}_{D*}, \tag{36}$$

but again for a defensible likelihood we need arrivals

$$\sum_i \gamma_{IC}^{pre} ICU^i_{pre*} + \gamma_U ICU^i_{W_r} + \gamma_U ICU^i_{W_D} + \gamma_U ICU^{i,1}_D + \gamma_U ICU^{i,2}_D \tag{37}$$

and departures

$$\sum_i \gamma_{IC_{W_r}} ICU^i_{W_r*} + \gamma_{IC_{W_D}} ICU^i_{W_D*} + \gamma_{IC_D} ICU^{i,2}_{D*} \tag{38}$$

The death rate in hospital is

$$X_{H_D} = \sum_i \gamma_{IC_D}(ICU^{i,2}_D + ICU^{i,2}_{D*}) + \gamma_{W_d}(W^i_D + W^i_{D*}) + \gamma_{H_d}(H^{i,2}_D + H^{i,2}_{D*}) \tag{39}$$

The care home death rate is given by

$$X_{G_D} = \gamma_{G_D} G^{i,2}_D \tag{40}$$

The testing data require

$$X_{S+} = \sum_{i=3}^{12} T^i_{S+} \tag{41}$$

(summation over ages 15 to 65) and

$$X_{R1+} = \sum_{i=1}^{17} T^i_{P+} \tag{42}$$

(summation from age 5 upwards and care home workers, but not residents). In Knock et al. there is one further stream for the Pillar 2 PCR data, but the model seems so crude that this stream can really only undermine inference and is better omitted.

B Statistical model and inference

This appendix details the priors used, likelihood and the inferential approach taken.

B.1 Priors

Parameters were optimized on a working scale, so that the dynamic model parameters were $\theta_j = h_k(\theta'_j)$ where θ'_j was unconstrained and k selects among 3 alternatives. h_1 was the identity, h_2 the exponential and $h_3 = a_1 + (a_2 - a_1)e^{\theta'_j}/(1 + e^{\theta'_j})$, which constrains the θ_j to the interval (a_1, a_2). The limits were generally set from the prior intervals given in Knock et al. (2020). Gaussian priors on the working scale were also applied, but except for t_0 these were vague, and their only purpose was to allow ready detection of any parameters that were not identifiable.

	ϵ	m_{chw}	m_{chr}	t_0	p_H^{max}	p_G	μ_{IC}	p_{IC}^{max}	$p_{IC_D}^{max}$	μ_D	$p_{W_D}^{max}$	$p_{H_D}^{max}$
h	2	2	2	1	3	3	3	3	3	3	3	3
a_1					0	0	.66	.14	.57	.66	.25	.36
a_2					1	1	1	.36	.77	1	.46	.56
μ_p	-.7	-12.7	-12.7	30	1.1	1.1	-1	-.2	0	-1	0	0
σ_p	2	2	2	10	2	2	2	2	2	2	1	1

h_2 was used for γ_G, $\gamma_{IC_{W_r}}$, γ_{W_r} and γ_{H_r}, with working scale mean vector $\mu_p = (-1, -2.75, -1.8, -2.37)$ and $\sigma_p = 2$ in all cases. The means were set from the Knock et al. (2020) estimates. The h_3 applied to the adaptive spline to yield $b(t)$ had $a_1 = 0$ and $a_2 = 0.1$. Note that a_1 was set to 0.8 for μ_D in the two Northern regions - this avoids the model trying to compensate for not representing hospital acquired infections by driving down the IFR too fast in the first wave, leading to overestimation of hospitalizations in the second.

B.2 Likelihood and data

The likelihood is constructed from negative binomial and binomial components. The binomial log likelihood is used for the serology and REACT PCR data.

$$l_b(p) = y \log p + (n - y) \log(1 - p) + \log n! - \log y! - \log(n - y)!$$

so

$$\frac{\partial l_b}{\partial p} = \frac{y}{p} - \frac{n - y}{1 - p}.$$

The negative binomial log likelihood is used for admissions, general ward and ICU occupancy and deaths.

$$l(\mu, \kappa) = \kappa \log \left(\frac{\kappa}{\kappa + \mu} \right) + y \log \left(\frac{\mu}{\mu + \kappa} \right) + \log \Gamma(\kappa + y) - \log \Gamma(\kappa) - \log y!$$

and so

$$\frac{\partial l}{\partial \mu} = \frac{y}{\mu} - \frac{y + \kappa}{\mu + \kappa}.$$

As discussed in the main paper, the likelihood based on independent negative binomial deviates is not justifiable for occupancy, and an alternative based on changes in occupancy, which can be modelled as independent, is more appropriate. If we model the ward (or ICU) arrivals as Poisson, and the departures as Poisson, then this daily change will follow a Skellam distribution, but that allows for no overdispersion. The difference between negative binomials (with common κ parameter) is a skewed generalized discrete Laplace distribution, but is computationally awkward. We therefore model ward and ICU arrivals and departures using overdispersed versions of the normal approximation to the Poisson, $N(\mu_1, k\mu_1)$ and $N(\mu_2, k\mu_2)$, with difference $N(\mu_2 - \mu_1, k(\mu_1 + \mu_2))$. Let $\sigma_0 = \mu_1 + \mu_2$ and $\alpha = (y - \mu_1 + \mu_2)$, where y is now the observed change in occupancy, then

$$l_d(\mu_1, \mu_2) = -\frac{\alpha^2}{2k\sigma_0} - \log(k\sigma_0)/2 - \log(2\pi)/2$$

and so

$$\frac{\partial l_d}{\partial \mu_1} = \frac{\alpha}{k\sigma_0} + \frac{\alpha^2}{2k\sigma_0^2} - \frac{1}{2\sigma_0}, \quad \frac{\partial l_d}{\partial \mu_2} = -\frac{\alpha}{k\sigma_0} + \frac{\alpha^2}{2k\sigma_0^2} - \frac{1}{2\sigma_0}.$$

A difficulty with applying this model directly is that hospital arrivals and discharges tend to have weekly pattern. This pattern shows up strongly in the ACFs and PACFs of occupancy first differences for some

regions, especially East of England, but is absent from the model. We therefore base the likelihood on weekly changes. Since the changes in occupancy carry no information on the level of occupancy, we also add the sum of daily bed occupancies as a final datum to be fitted, treating this as close to Poisson (by setting κ to a very high constant).

For the total daily hospital admissions data and the care home deaths data we retain the negative binomial model, with the respective κ parameters free to be estimated. Some overdispersion to deal with likely model mismatches in these components seems pragmatic. For the hospital deaths we set $\kappa = 2000$, which gives a likelihood very close to Poisson. There seems no legitimate reason to expect overdispersion here if the model is at all fit for purpose.

B.3 Estimation and inference

Given smoothing parameters, $\boldsymbol{\lambda}$, and overdispersion parameters we find the posterior parameter modes

$$\hat{\boldsymbol{\theta}} = \underset{\boldsymbol{\theta}}{\text{argmax}} \;\; l(\boldsymbol{\theta}) - \frac{1}{2}\boldsymbol{\theta}^{\mathsf{T}}\mathbf{S}_\lambda\boldsymbol{\theta} - \frac{1}{2}\sum_j(\theta_i - \mu_j^\theta)^2\nu_j^\theta, \text{ where } \mathbf{S}_\lambda = \sum_j \lambda_j \mathbf{S_j}, \tag{43}$$

l is the log likelihood and the $\mathbf{S}_j$ are fixed positive semi-definite matrices imposing the spline smoothing penalty. μ_j^θ and ν_j^θ are prior means and precisions. The precisions may be zero, and are for all spline coefficients. Quasi-Newton optimization was used to find $\hat{\boldsymbol{\theta}}$ (see e.g. Nocedal and Wright, 2006). Let $\mathbf{H}_\lambda$ denote the Hessian of the negative of the penalized log likelihood given in (43), evaluated at $\hat{\boldsymbol{\theta}}$ (it is obtained by finite differencing the computationally exact gradients). Then we have the large sample approximation to the posterior

$$\boldsymbol{\theta} \sim N(\hat{\boldsymbol{\theta}}, \mathbf{H}_\lambda^{-1}). \tag{44}$$

The smoothing parameters were obtained using the approximate marginal likelihood maximization method of Wood and Fasiolo (2017), which alternates optimization of (43), given smoothing parameters, with updates of all smoothing parameters, given $\hat{\boldsymbol{\theta}}$

$$\lambda_j \leftarrow \frac{\text{tr}(\mathbf{S}_\lambda^- \mathbf{S}_j) - \text{tr}(\mathbf{H}_\lambda^{-1}\mathbf{S}_j)}{\hat{\boldsymbol{\theta}}^{\mathsf{T}}\mathbf{S}_\lambda\hat{\boldsymbol{\theta}}}\lambda_j.$$

At each step of this iteration the overdispersion parameters were obtained by maximum likelihood estimation, given the current model predictions. Uncertainties for incidence and R trajectories were obtained from (44) by the delta method (applied on the log scale).

C Selection pressure and lockdowns

Here we sketch a simple mathematical argument implying that lockdown and social distancing measures could in principle remove the most obvious selective advantage for milder disease. The point of this is not to suggest that this has actually happened, but simply to point out a very basic mechanism by which it could happen. This analysis in turn suggests that the opposite effect, of lockdown and social distancing promoting milder disease, seems rather unlikely, a priori.

Letting R denote the number of new infections caused by an existing infection, we write $R = \alpha MV$ where V denotes level of viral shedding, M denotes number of contacts and α is a constant of proportionality. Assuming that higher viral shedding tends to correlate with more serious disease, then in normal circumstances we can expect a negative relationship between M and V, as more serious illness somewhat incapacitates the host, so that they are likely to reduce social contact (for example they take

to bed). There is a further possibility in the case of a disease like SARS-CoV-2 that very serious disease then increases contacts again as hospitalization becomes necessary. The selection pressure that this exerts in the absence of strict infection control is obvious and we therefore neglect this aspect here.

Therefore let $M(V)$ be a continuous strictly positive monotonically decreasing function of V, so that

$$R = \alpha M(V)V,$$

and

$$\frac{\mathrm{d}R}{\mathrm{d}V} = \alpha M'(V)V + \alpha M(V).$$

If $\mathrm{d}R/\mathrm{d}V > 0$ then increased V increases fitness (the reproductive rate, R), so selection pressure is for increased V. If $\mathrm{d}R/\mathrm{d}V < 0$ then the pressure favours reduced V. Hence if

$$M'(V) > -\frac{M(V)}{V}$$

selection favours increasing V, while decrease is favoured when the inequality is reversed. Recall that $M'(V) \leq 0$. So whenever $M(V)$ declines sharply enough with increasing V, selection acts to decrease V, while less steep decline tends to select for increased V.

We have stated this simple argument generally to emphasise that it is not reliant on particular parametric assumptions about $M(V)$, but simply on how M declines with V. A problem with any strategy aimed at reducing asymptomatic spread is that it reduces mixing at low V disproportionately relative to mixing at high V (since at high enough V illness already limits mixing, both directly and as a result of public health advice). This in turn moves the system towards the conditions favouring higher V. Fig 1 shows an illustrative example.

References

Dennis, J. M., A. P. McGovern, S. J. Vollmer, and B. A. Mateen (2021). Improving survival of critical care patients with coronavirus disease 2019 in england: a national cohort study, March to June 2020. *Critical care medicine 49*(2), 209.

Diekmann, O., J. A. P. Heesterbeek, and J. A. Metz (1990). On the definition and the computation of the basic reproduction ratio R0 in models for infectious diseases in heterogeneous populations. *Journal of mathematical biology 28*(4), 365–382.

Funk, S. (2020). *socialmixr: Social Mixing Matrices for Infectious Disease Modelling*. R package version 0.1.8.

Knock, E. S., L. K. Whittles, J. A. Lees, P. N. Perez Guzman, R. Verity, R. G. Fitzjohn, K. A. M. Gaythorpe, N. Imai, W. Hinsley, L. C. Okell, A. Rosello, N. Kantas, C. E. Walters, S. Bhatia, O. J. Watson, C. Whittaker, L. Cattarino, A. Boonyasiri, B. A. Djaafara, K. Fraser, H. Fu, H. Wang, X. Xi, C. A. Donnelly, E. Jauneijaite, D. J. Laydon, P. J. White, A. C. Ghani, N. M. Ferguson, A. Cori, and M. Baguelin (2020). Report 41: The 2020 SARS-CoV-2 epidemic in England: key epidemiological drivers and impact of interventions. *Imperial College London*.

McKeigue, P. M., D. McAllister, D. Caldwell, C. Gribben, J. Bishop, S. J. McGurnaghan, M. Armstrong, J. Delvaux, S. Colville, S. Hutchinson, et al. (2021). Relation of severe COVID-19 in scotland to transmission-related factors and risk conditions eligible for shielding support: REACT-SCOT case-control study. *BMC Medicine*.

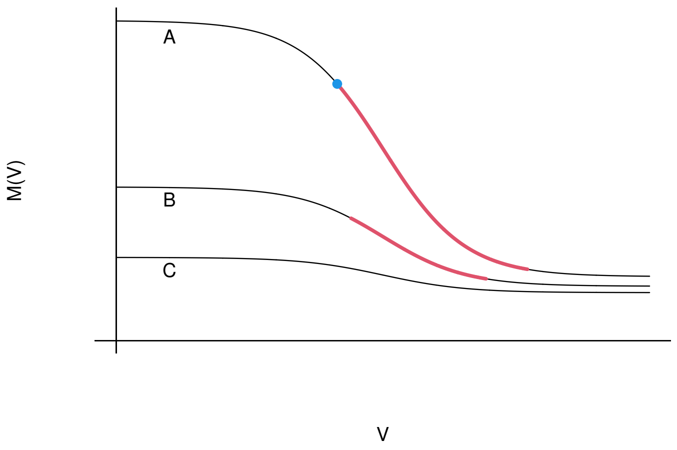

Figure 1: Illustrative mixing, M, versus viral load, V, functions, for a disease where infections may range in severity from asymptomatic with low load to severe enough to prevent normal mixing, with higher load. Curve A shows M against V without social distancing and other blanket reductions in mixing. The thick red curve is the region where $M'(V) < -M(V)/V$ so that reducing V increases fitness. The blue dot marks the point of highest fitness over the V range shown. B shows an $M(V)$ in which low V mixing has been substantially reduced by blanket reductions in mixing. High V mixing was already low, so that there is little scope for further reduction. For this curve the red region, in which lowering V confers a selective advantage, has been reduced. The highest fitness point over the range shown is now at the highest point in the range, although the lower V end of the red region is still a local optimum. C shows a curve where the reduction in asymptomatic transmission has been so successful that increasing V always increases fitness. Note that strict self isolation only of symptomatic cases can actually steepen the curve relative to the 'no interventions' case.

Mossong, J., N. Hens, M. Jit, P. Beutels, K. Auranen, R. Mikolajczyk, M. Massari, S. Salmaso, G. S. Tomba, J. Wallinga, J. Heijne, M. Sadkowska-Todys, M. Rosinska, and W. J. Edmunds (2017, November). POLYMOD social contact data.

Nocedal, J. and S. Wright (2006). *Numerical Optimization* (2nd ed.). New York: Springer Verlag.

RECOVERY Collaborative Group (2020). Dexamethasone in hospitalized patients with covid-19—preliminary report. *New England Journal of Medicine*.

Wood, S. N. and M. Fasiolo (2017). A generalized Fellner-Schall method for smoothing parameter optimization with application to Tweedie location, scale and shape models. *Biometrics 73*(4), 1071–1081.
