# Supplementary material for: Was R < 1 before the English lockdowns? On modelling mechanistic detail, causality and inference about Covid-19
Source: PLoS One. 2021 Sep 22;16(9):e0257455. doi: 10.1371/journal.pone.0257455 (PMC8457481; doi:10.1371/journal.pone.0257455)
Supplement: Attachment — Submitted filename: response.pdf [file pone.0257455.s003.pdf]

Response to review comments

We would like to thank the referees for their careful reading of the paper. While we do not agree with a minority of their comments, as detailed below, others have substantially improved the paper, and we are grateful for the thorough job that they have done.

Since the response to the individual comments is necessarily rather lengthy, we summarise the substantial changes made to the paper in response to the referee comments here:

1. We present the results of sequential relaxation of the Knock et al. model assumptions, as suggested by the referees, in figure 6.

2. We added day/month scales to the main reconstruction plots as well as the dates of interventions preceding full lockdown in March 2020.

3. We added 2 references quantifying negative consequences of lockdowns and a supplementary material section on possible selection pressure effects of lockdown, other social distancing and track-and-trace measures.

4. We modified the proportion arriving pre-tested function in the light of finding some (rather limited) data on this.

5. We added uncertainties to figure 3 (credible intervals and in some case prediction intervals).

6. We have made most, but not all, of the wording changes suggested by the referees in the interests of a more balanced presentation. However for a minority of these suggestions we felt we were being asked to be more balanced between correct and incorrect, and these we have not followed.

In addition we made two further changes not based on referee comments.

1. Since original submission several results have been published on direct incidence reconstruction from statistically unbiased data sources. They concur with our results, and this is now discussed in a paragraph and figure 7 in the discussion.

2. One panel of figure 6 shows a simple sensitivity analysis on the smoothness assumption on $b(t)$.

3. Knock et al was in fact accepted for publication in *Science Translational Medicine* 2 days before we received the referees' reports and decision letter from PLOS. It had been submitted in mid April, 2.5 months after we supplied Knock et al. with our results. It was published on line the next week, but we only located it after responding to the referees comments. Since the revised paper was returned to us for formatting reasons, we have now added a paragraph in the discussion discussing what has changed in the accepted version of the paper.

Editors Comments

As required we have followed the plosone guidelines and used the supplied latex template for manuscript preparation. To facilitate review, we have used the previous style for the version showing changes, however, and we have left the figures in the pdf, for the same reason.

There was a request to provide a Table 1. This seems to be a misunderstanding, the only reference to table 1 is when citing a report on R estimation - the table being in the report.

I echo one of the reviewers in the suggestion of putting the authors claim in a more objective fashion, in order to provide the readers with a balanced counterpoint to [Knock et al., 2020], rather than a mere criticism of their work, especially in light of the fact that both studies make simplifying assumptions that might bias their claims.

Where possible, we have tried to follow the reviewers suggestions, as detailed below. However, given that our work is fundamentally an assessment of Knock et al. which attempts to relax some of its stronger assumptions, while correcting clear discrepancies with the cited literature and components of the likelihood that are impossible to justify or simply wrong, it would not be correct for us to present the assumptions as equally valid alternatives.

In this view, another reviewer requires more discussion about the authors' assumptions and the criticism of [Knock et al., 2020] ones. I agree with the reviewer that a more detailed and balanced discussion is required. In my opinion, as well as according to many other scholars, it is very hard to disentangle the single effects of different measures on the spreading of COVID-19, and such a study is strongly dependent on the simplifying assumptions made to carry out the study. Hence, I think that the proposed manuscript would greatly benefit by the analysis of a more balanced viewpoint, highlighting strengths and weaknesses of both set of assumptions, and, possibly, to the consequences that such assumptions may entail in both studies.

We agree completely that causal inference about Covid measures is very difficult (although falsifying causality can often be much easier, of course), and this is indeed the main point that we want to make. The fact that *relaxing* the assumptions of the Knock et al. model reverses the conclusions about timings relative to lockdown clearly indicates that this model can not be used to make strong causal statements. Our secondary point, is that even *if* you believe the basic structure of the Knock et al. model to be appropriate for inference, then relaxing the very restrictive model for the NPI effects for something more data driven, and moving the key progression rates towards the literature values cited by Knock et al. actually suggests $R < 1$ before lockdown.

Reviewer 1

The manuscript contains a sophisticated compartmental model which allows for capturing the course of COVID-19 pandemic in England. In particular, by fitting the model to different epidemiological indicators such as the number of fatalities, hospitalizations or the evolution of test positivity, the authors are able to infer the actual epidemic incidence, i.e. the number of daily new cases, and study the evolution of the effective reproduction number, which is a pivotal indicator to quantify the stage of an ongoing outbreak.

The analysis presented by the authors represents a critical assessment of a report published by an independent research group in December 2020 addressing the same problem [Knock et al. 2020]. There, it is claimed that lockdown measures during the first epidemic waves in England were essential to reduce the effective reproduction number below one and, as a consequence, observe a decrease in the daily incidence.

The findings here contradict the claims made in [Knock et al. 2020] and show that the epidemic incidence started decreasing before full lockdown measures were implemented in England. The discrepancies between both results mainly arise from three factors: i) the choice of a shorter generation time interval, ii) the correction of the typical time elapsed from the onset of symptoms to hospitalization and iii) the continuity of the function reflecting the impact of non-pharmaceutical interventions. The mathematical framework proposed here is solid and described in depth and the methodology followed by the authors is clearly explained, which make the manuscript scientifically sound.

This summary is broadly correct, however it gives the impression that the generation/serial interval is key, which is not the case (see new figure 6). It is also not the case that it is simply a different 'choice'. Knock et al. have a serial interval of 8-9 days, which is well above the literature range of 5-6, and their model structure would make this even longer if they had used the incubation period and hospitalization time given in the literature they cite as sources for these. In addition to the correction of the hospitalization time the correction of the incubation time also matters. Finally, the degree of continuity of the $b(t)$ function is not the major issue, it is the flexibility it is allowed that is key (we could have obtained the same results with a piecewise linear $b(t)$ of sufficient flexibility, although there is no

good reason to do so). Our model is set up to allow a far wider range of function shapes, and hence to allow the shape of $b(t)$ to be driven by the data, rather than by prior assumption. We have slightly expanded the first paragraph of the section *Relaxing the model assumptions* to make this clearer.

My main criticism with the current manuscript concerns its writing style. In this sense,I think that the authors should tone down some parts of the manuscript to make their critical assessment more objective. I would also encourage the authors to keep their analysis at the scientific fundamental level rather than discussing further political implications. Some examples of parts which, in my opinion, should be rewritten are:

Page 2: The comments about the media attention of the reports published by the team from Imperial College London or their relevance for policy-making lie out of the scope of a scientific assessment of the model.

The referee is absolutely right that the policy impact and media attention have no bearing on our scientific assessment of the report's contents. However they do have a major bearing on why such an assessment matters, and, we would argue, on the standards of scientific rigour that it is appropriate to be working at. Our understanding of PLOS publication policies is that these comments in no way contravene them. That said, the fact that the report was accompanied by press release material is not really interesting unless it got press coverage. So we've removed the comment about press release material and included instead a brief mention of actual coverage.

Page 3: "Knock et al. do not report such checks, showing only the outputs of filtering" I think that this does not provide any valuable information for the discussion made in the paragraph.

Yes the wording could have been better. We think that stating what Knock et al. show is important, as it has a direct bearing on the strength of the evidence that they have presented. But we have re-worded to avoid the judgemental wording involved in using the word 'only', and instead providing only a neutral statement of what they did and did not show.

Pages 4-5: "The model captures many features in impressive detail, but several aspects are not modeled". The enumeration following this sentence describes very exhaustively many limitations of the model introduced in [Knock et al. 2020], most of them are not introduced in the model proposed here by the authors either. As in any theoretical model, some assumptions should be made to reduce the number of equations and not to end up with an unmanageable huge parameters' space. As the submitted manuscript does not incorporate most of these logical limitations of theoretical frameworks, I do not see the point of including such exhaustive description.

The referee is right that since we failed to make clear why these points matter to the case we make, they might look like sniping. We have now rectified this, with the following paragraph, which also explicitly states that part of our reason for giving the list is to draw attention to the limitations of the model that we use.

> Any biological model for a complex system necessarily makes many simplifying assumptions, often without substantial detriment to statistical inference within the range of the data being modelled. However causal inference based on statistical methods puts much heavier requirements on the model, since it is then required to extrapolate. Counterfactual statements made using a model are of this causal character, and in the current case require the model to behave essentially as a mechanistic representation of reality (since we know of no causal inference strategy that could alleviate the effects of mis-specification in this sort of model, and Knock et al. do not report any). Given this requirement for high mechanistic accuracy, any of the preceding omissions may be problematic. We note also that although we do not seek to extrapolate in this paper, most of these points will have some impact on our results. The hospital acquired infection issue makes it particularly difficult to exactly match hospital data with the model, for example.

Page 6: "It is hard to understand this choice, unless it was made to avoid particle depletion problems in filtering". This statement seems very subjective and can be expressed in other words or left to the interpretation of the reader.

We have changed this to a more balanced wording: "Although such a choice will reduce particle depletion problems in filtering, it is not easy to justify as a statistical model." To go further would be to fail to communicate that this is the wrong model, and to fail to communicate to readers unfamiliar with particle filtering that the choice does have a purely computational advantage.

Page 7: "While not ideal, this is a less wrong assumption"

We have removed this statement, having located some published information on this topic and modified the model accordingly, citing the reference.

Page 9: "Given the ambitious nature of the fitting task, it seems reasonable to view the results as useful in the 'all models are wrong, but some are useful' sense."

We have now included the attribution to indicate that we are quoting the statistician George Box.

Page 12: "We conclude that the model and inference of Knock et al. do not form any sort of reasonable basis for making counterfactual statements about how many people would have died if lockdown had occurred at a different time"

While our conclusion is fundamental, and we do not see how we could honestly say otherwise, the referee is right that the language went too far. In particular the 'any sort of' qualifier is not needed, and we have removed it.

Concerning the contents of the manuscript, I have some suggestions which, in my opinion, would make the result presented by the authors more robust: Page 2: For the sake of readability, it would be very useful to include some references supporting how lockdowns are detrimental to the evolution of the pathogen or explaining the economic side effects observed in many countries.

We have added a section to the supplementary material on selection pressure, and references to a UK government report and a UNICEF report on lockdown impacts. There is more that could be added, of course, but while it is important to briefly set the context of the work, we do not want this to become a major focus.

The expression for the time derivative of the sensitivities of each variable with respect to the different parameters is valid when the parameters θ are constant and do not depend on time. Nonetheless, the function $b(t)$ is time-dependent and, if I am correct, this expression should not be valid in this case. Please clarify it.

We use a basis expansion for $b(t)$, the coefficients of which are fixed, hence the expressions are correct for the coefficients of $b(t)$. We have included a parenthetic comment explaining this in the paper.

I think the manuscript would clearly benefit from a figure showing explicitly how the evolution of the effective reproduction number depends on the generation time interval chosen or the time to hospitalization as reflected by the authors in Page 9.

This is a very helpful suggestion. We have now included this in new figure 6, which makes matters much clearer we think.

I would avoid mentioning seasonality as a crucial confounding factor invalidating the counterfactual scenarios proposed in [Knock et al. 2020] because, in the short time windows analyzed in the latter report, one cannot expect substantial underlying changes in the weather conditions.

The expectation of no substantial change is not born out by the data. See fuller answer to referee 2 on this. Note that we focus on weather to highlight that un-modelled confounders certainly exist. We don't, and can't, know how crucial it is relative to much more difficult to measure confounders, such as behavioural changes in direct response to the perceived health threat, independent of government mandate.

The section describing the computation of the effective reproduction number should be extended to better explain the significance of this indicator. In particular, within the different alternatives explored

in the literature (see [Gostic et al. PLoS Comput Biol 16(12): e1008409 (2020)] for a further review on the topic), the authors compute the reproduction number as the expected number of contagions that an agent becoming infectious at time t will make throughout his/her infectious period if the conditions of the system remain immutable.

We have now defined R conceptually where it is first introduced, after the SEIR, and briefly pointed out that extra assumptions are required to obtain an instantaneous measure.

In this sense, the explanation of the dependence of the effective reproduction number after lifting lockdown measures in page 9 is not valid if one resorts to this definition. Providing the model does not divide the population into key workers and locked-down individuals either, I think that the authors could get rid of this discussion.

There appears to be a misunderstanding here, caused by sloppy writing on our part. The discussion is about what happens after the *introduction* of the first lockdown, not after its lifting. We have now re-worded to make this clear. The discussion indeed makes no sense as a discussion of what happened after lockdown was lifted.

The mechanism we discuss explains the basic arithmetic that must apply to the average number of new infections per infection in a real population that is in fact stratified into 'lower R' and 'higher R' strata. On the basis of aggregated data from such a system, all we see is that R shows an initial drop followed by a partial recovery to the R level in the 'higher R' stratum. A model without the relevant strata can match such data by allowing R to drop and recover, but of course is reproducing the effect phenomenologically, not mechanistically. To try to make this clearer we have added the following to the paragraphs where this is discussed.

> Although the simple arithmetic mechanism underlying this effect results from having locked down and key worker strata, we only observe aggregate data, reflecting the change in R, but not what causes it. The model also deals only with populations aggregated over the two strata, but can still capture the change in R apparent in aggregate data, if $b(t)$ is flexible enough. However, the piecewise linear $b(t)$ of Knock et al. is not flexible enough in this regard.

Finally, to obtain the reproduction number at the national level, the authors should weight the individual reproduction numbers by the newly infected individuals in each of the regions rather than with the infected population.

This does not seem mathematically correct. Following Knock et al., the model is set up so that all infectives in the stage have the same expected time remaining as infectives, irrespective of how long they have been in it that state already. So anyone still in the stage has exactly the same expected number of future infections caused as someone newly entering the stage: new infectives and old infectives are indistinguishable. The referee's suggestion would be the better approximation for a fixed length stage, but not when the duration follows an exponential distribution. However we have changed the wording of the R figure caption to be clear exactly what average is being used.

Reviewer 2:

The manuscripts provides a revision of the study by Knock et al. (2020). The authors highlight various caveats and adapt the model accordingly. The main finding is that, in contrast to Knock et al., the reproduction number is found below one before the implementation of the two lockdowns in England. In this sense, the authors then question whether the lockdowns were the main driver to control SARS-CoV-2 in England.

Our view is that we have identified serious shortcomings in the original study that substantially undermine the conclusions made using it. We think that the shortcomings are more serious than 'various caveats' might imply. The original study uses an incubation time and time to hospitalization that are

substantially shorter than the figures actually given or implied by the studies they cite as sources, while the serial interval used is longer than any value given in the literature. These unexplained departures from the literature, along with the very restrictive choice of breakpoints in the $b(t)$ function, completely drive the result that R was substantially above 1 before the first lockdown. And that timing result is the essential component of any argument that lockdown is the key causal control factor for infections. Without that the remaining results can not be valid.

The approach the authors take is well motivated and scientifically sound. The authors highlight various shortcomings of the study by Knock et al. and adapt different aspects of the model. Unfortunately, due to the various changes made, it is not possible to isolate the aspect that lead to the different result. It may be due to the shorter generation time, the adapted time from symptom onset to hospitalisation or the different functional form of R(t). I guess that the fit is very costly to perform, but I strongly recommend to incrementally adapt the model by Knock et al., which would allow to isolate the impact of the various changes made.

This is a really helpful suggestion. Actually the statements about the speed up in fitting that we make in the introduction are not exaggerated, and fitting takes less than 3 hours using 7 cores of a 10 year old desktop workstation. We have therefore presented a sequence of runs, in which we first relax the assumptions on $b(t)$ but leave the timings the same, then move the serial interval into the range suggested by the literature, and finally change the incubation period and hospitalization times to those actually stated in in the papers cited by Knock et al. We also added a run in which we deliberately undersmooth, to investigate sensitivity to the smoothness assumption on $b(t)$. These are now shown in figure 6, and discussed in the results section.

The inferred reproduction number R(t) starts to decrease almost immediately (Figure 5). How do the authors explain this early decrease? I guess no NPIs were in place back then and mobility still showed no reduction. In this sense, please explicit in a table when the reproduction number actually was below one. It seems that during the first wave the reproduction number in London was below one surprisingly early. Additionally, I strongly recommend the authors to work with dates instead of integer numbers for the day of the year. It would improve the readability of the manuscript a lot.

We have added month-day scales to the top axis of figures 4-6, while retaining day of year scales on the bottom axis. This enables ready comparison with the other plots where the date scales would be cumbersome.

We have also added the dates of measures in place before lockdown (previously only mentioned in the discussion) to plots 4-6 and described what those were in the caption of figure 4.

Note that google mobility data, for example, show a very pronounced changes well before lockdown:

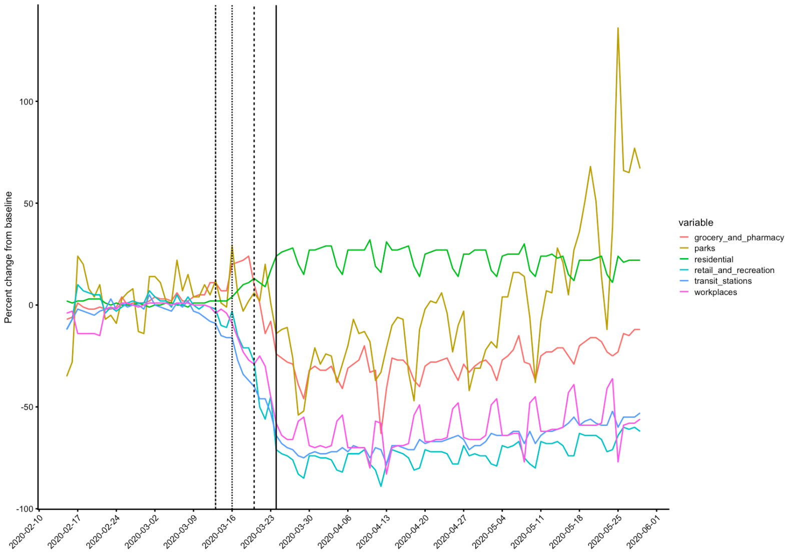

Given the great difficulty of properly using opportunistic data of this sort, we are very reluctant to encourage informal interpretation of them by including them in the paper. However, since we submitted the paper, statistically well founded measurements of incidence have become available supporting our paper's results, and we have now included these in the discussion and figure 7.

In the manuscript here, the model consists of regular ODEs, whereas Knock et al. worked with a stochastic model. The authors write: "The neglect of stochasticity in the state equations seems likely to be a minor issue here, relative to the other approximations made in the model." In principle I agree with this statement. However, looking at the results (Figure 3) the peak in care home deaths is anticipated with respect to the data in all regions. One explanation could be the lack of stochasticity that may delay infections in care homes due to the modular contact matrix. Another factor could be the different initialisation the authors choose as it is pointed out in the manuscript.

The Knock et al. results (their Figure S7) shows a similar pattern, but slightly less pronounced. However they appear to be showing the distributions of filtered states rather than posterior predictive plots, and as discussed in the paper filtering will pull the states towards the data, even if this is quite improbable under the model. The referee is right that stochastic effects could lead to this sort of delay, and the filter may well be selecting transitions corresponding to such an effect. However in reality the effect would apply at the individual home level, so that summing over multiple homes would require a very improbable set of events to see a marked effect in the aggregate.

One problem with the care home model is how crude it is relative to the hospital model, and how extremely limited the data are on care-home mixing (the POLYMOD survey has 7 participants over 75 and none over 80, and it seems highly unlikely that care home mixing was really controlled with the same $b(t)$ as the general population). We think that these issues are likely to be more important than the deterministic versus stochastic differences.

In Figure 3, one can see that occupancy and admissions generally peak earlier than the data and decrease faster. How do the authors explain this mismatch? Furthermore, why is there no uncertainty range indicated in Figure 3? Is it too small to be visible? However, this would not be consistent with the uncertainty ranges presented for the incidence (Figure 4) and the reproduction number (Figure 5).

These features are also present in the Knock et al fits (see their Figure S7). The absence of nosocomal infection in the model makes the later peak in the real data almost inevitable. We also suspect that there is less pressure to discharge on the down-sweep. We slightly modified the wording to draw the reader's attention to the fact that the hospital data include hospital acquired infections, which are not modelled explicitly.

The figure plots were the posterior modes for the various quantities plotted (as stated in the caption). A posterior mode is not uncertain - it is a fixed parameter of the distribution characterizing uncertainty. We plotted this way for clarity and to emphasise where the model mis-fits. However we have now instead plotted (nominal) 95% credible bands for the various quantities. In addition we have plotted 95% prediction intervals for the deaths and hospital admissions. We have not added these for occupancy, since occupancy is not modelled directly, for the reasons given in the paper (we model differences), and prediction bands are therefore rather technically awkward to compute. We have also not plotted prediction bands for the test data - such bands are easy to compute, and cover the data as expected, but are exceptionally ugly as a result of the wide variation in sample sizes and hence interval width, between samples.

In the discussion, the authors mention a sensitivity analysis they performed. In particular, they considered different time to hospitalisation, generation time and likelihood for occupancy. Please show these results in a supplementary. Because comments like "... $R = 1$ a little later, but still before lockdown" are not very helpful for the reader. Indicate how much later and when if you already performed the fit.

We have now done this - see figure 6.

The authors criticise many times that Knock et al. did not isolate the impact of seasonality and NPIs. Furthermore, you claim that this invalidates the counterfactual scenarios. If the counterfactual scenarios consisted of shifting the time series by months this would certainly be true. However, Knock et al. consider a maximal shift of two weeks. Do the authors truly believe that the meteorological change in two weeks invalidates the conclusions of Knock et al.?

Our point is the general one about what is required for counterfactual validity in statistical casual inference, with weather being only the most obvious example confounder. That said, in the UK week to week variability in weather is every bit as extreme as its stereotype, particularly in Spring and Autumn. Here is the daily temperature and rainfall record for 2020 from the UK Met office (full lockdown was March 24th).

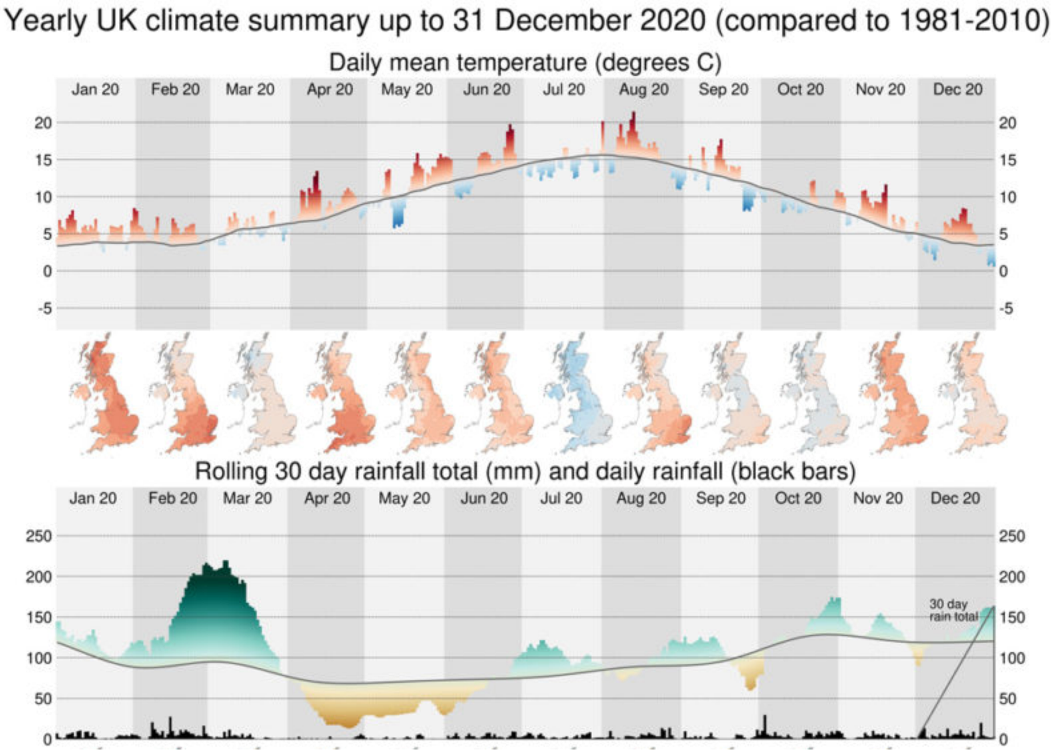

https://www.carbonbrief.org/met-office-a-review-of-the-uks-climate-in-2020

. . . 2 weeks is clearly a long time in UK weather. However even if the weather were not so rapidly varying, the informal reasoning that 'it's only 2 weeks' would not be right - the confounding affects the whole course of the inference about $b(t)$, and with so few change points allowed in the Knock et al model in the first half of 2020, weather effects on inference can not be localised to during that period: the whole shape of the inferred $b(t)$ is impacted. Further, the figures for 'lives saved' involve integration over months, not just weeks, and through that whole period there is confounding.

The authors criticise that Knock et al. did not model within hospital transmission. Furthermore, they claim within hospital transmission account for a quarter of cases in both waves and cite a health service journal. Is this with respect to the reported cases or do the authors believe this also holds for infections in general? Be more specific whether you refer to reported cases, which will be strongly biased towards within hospital transmission. Furthermore, making such claims based on a reference to a journal does not seem appropriate for a scientific article.

Good point, it's hospitalized cases that we meant, and this is now stated explicitly. The figures are backed up with a scientific journal article in press in BMC medicine, and we have now cited the publicly available NHS data supporting the reports (although first wave data does not appear to be publicly available).

The authors point out that including data regarding the number of tests undermines the analysis and should therefore not be included. However, I do not understand whether you actually did fit testing data or not. Later in the manuscript you explain the different assumption made regarding the pre-tested rate. This led me believe that you actually fit to the testing data. Please clarify this point. Also you should be consistent, if you truly believe that it undermines the analysis then don't include it.

The data we do not believe should be fitted is the proportion testing positive in all NHS testing. The discussion the referee refers to is about the proportion of people arriving at hospital pre-tested. The description of this has been modified a little in the light of finding a new reference (see response to referee 1), so hopefully the confusion will not arise again. We do fit the proportions testing positive in the statistically valid samples provided by the REACT study.

A more general remark is regarding the writing of the manuscript. Various parts are not written with the objectivity a scientific article requires. For example, criticising the approach of Knock et al. for not modelling the differences between men and women seems really far fetched.

We now cite a prominent example of one of the many papers reporting large male - female differences.

Similarly, on page 6, you refer to a reasonable model. Rewrite this such that it is less judgemental. An example would be "a more accurate/better approach".

While we have toned down the other cases where the referees have identified overly strong wording, we think that in this particular case it is important to emphasise that the formulation is completely flawed. We would be very disappointed to find this likelihood in a student project, let alone in a report likely to have the serious real world implications of this one.

The same comment also holds for other parts of the manuscript: Page 7: "Less wrong assumption . . . ". Change it to better/ more realistic assumption.

Yes, we have removed this and modified the description in the light of the reference now given allowing slightly better justification of what we did.

Page 2: ". . . was accompanied by substantial press release material". Why is this relevant?

This is a fair point, we wanted to point out that the report had substantial impact, but our comment of course implies nothing of the sort. It has now had substantial press coverage, and we have mentioned that instead.

Page 1: You insinuate that the lockdown lead to a substantial loss of life due to the caused economic hardship. A scientific article is not the place to make insinuations. Referencing a pre-COVID study to back up a claim about the impact of lockdown is very vague and implicit. If you want to make such

comment find a study that actually treats the impact of lockdowns and aims at quantifying the non COVID related loss of life.

We wonder if the referee wants to suggest that we were somehow being deliberately underhand? We stand by what we have written. Lockdowns inevitably cause economic hardship and exacerbate inequality and in the UK there is a very strong association between economic hardship/inequality and early death. These are simply facts that deserve *some* attention in the discussion of whether the measures taken have been proportionate. If we wait until there is firm evidence of early deaths via this pathway, it is too late to prevent any of them. The argument that the most substantial review of the recent historic link between economic hardship and health outcomes in England is irrelevant because it predates covid is a very odd one, and we can not imagine another scientific topic to which such an argument would be applied and be viewed as reasonable. It seems a little bit akin to arguing that paleo-climate studies are irrelevant because they predate *enthropogenic* climate change, for example.

However we have now referenced a UK government study and a UNICEF study for South Asia in the following qualification of the original sentence.

> In England economic hardship and inequality are associated with very substantial loss of life, as reviewed in detail in Marmot et al. (2020). Whether the lockdowns will in fact have the same effects as previous hardship and inequality can not be stated with certainty, given that the well documented existing effects occur over longer timescales than have yet elapsed since the start of the epidemic. However it would seem irresponsible to simply assume no impact, and government assessments for the UK (DHSC, 2020) anticipate substantial effects, while UNICEF has already documented evidence for losses well in excess of direct covid life year loss in parts of south Asia (Bhutta et al. 2021).

We could expand this much further, but would rather not, since, although it is important to set the societal context of the science, it is not the main focus of the paper.

Page 12: You write "We conclude that the model and inference of Knock et al. do not form any sort of reasonable basis ... ". I understand your criticism of their approach, but saying that it does not support any reasonable basis is an exaggeration.

We have removed 'any sort of', which was obviously a silly thing to say. We have also explained our reasoning more fully

> Even without the preceding general problems, there is the specific problem that lockdown can not have caused R to drop below one if this event preceded lockdown, but the counterfactual statements rely on such a causal link.

References

Knock, E. S., L. K. Whittles, J. A. Lees, P. N. Perez Guzman, R. Verity, R. G. Fitzjohn, K. A. M. Gaythorpe, N. Imai, W. Hinsley, L. C. Okell, A. Rosello, N. Kantas, C. E. Walters, S. Bhatia, O. J. Watson, C. Whittaker, L. Cattarino, A. Boonyasiri, B. A. Djaafara, K. Fraser, H. Fu, H. Wang, X. Xi, C. A. Donnelly, E. Jauneijaite, D. J. Laydon, P. J. White, A. C. Ghani, N. M. Ferguson, A. Cori, and M. Baguelin (2020). Report 41: The 2020 SARS-CoV-2 epidemic in England: key epidemiological drivers and impact of interventions. *Imperial College London*.
