# Supplementary material for: Was R < 1 before the English lockdowns? On modelling mechanistic detail, causality and inference about Covid-19
Source: PLoS One. 2021 Sep 22;16(9):e0257455. doi: 10.1371/journal.pone.0257455 (PMC8457481; doi:10.1371/journal.pone.0257455)
Supplement: Attachment — Submitted filename: response-2.pdf [file pone.0257455.s004.pdf]

Response to 2nd review comments

Given that timeliness is really quite important for this topic, we are disappointed at the further delay on top of the 4.5 months that the paper has so far been in review, particularly since the comments do not affect the paper's main scientific conclusions. That said we have thoroughly considered all the points raised and responded to them as detailed below. The main changes are:

1. Mention of the interventions preceding full lockdown (and what full lockdown added) in the caption of Fig 1 - previously these were only mentioned later.

2. Expansion of the second paragraph of the introduction to address referee 2's comments on possible lockdown impacts.

3. More careful description of R where it is first defined, as suggested by referee 1.

4. Adding numerical confidence intervals on the first time of $R < 1$ to Fig 6 in response to referee 2.

5. Explicit mention of the first incidence peak timing in Fig 7 and plotting of the corresponding R estimates in response to referee 2.

6. A correction to our description of the results in Knock et al. (2021): they only have $R < 1$ before lockdown for London, not the 2 other regions we had initially thought.

We hope that it is possible for the paper to now be accepted quickly: clearly this is a topic where every week of delay diminishes the work's impact, and allows the claims in Knock et al. to propagate without challenge in the peer review literature. The scientific evidence base is only strengthened by proper timely scrutiny. If the evidence supporting full lockdowns is weak then it is important that this is acknowledged. If the evidence base is strong then it is only strengthened further if it is clear that studies constituting that evidence are being carefully examined and not simply accepted wholesale.

Reviewer 1

The authors have done an exhaustive work to address the comments raised by both referees and the revised version of the manuscript is much more solid and scientifically rigorous. Consequently, I think that the manuscript is now suitable for publication.

Nonetheless, I have a few minor comments regarding the modifications introduced by the authors:

- In my first revision, I thought that the authors were computing the effective reproduction number at time t as the number of contagions made by a newly infected individual during his/her infectious period. In case that the authors define the effective reproductive number at time t as the number of contagions made by an existing infectious individual at time t, which corresponds to the instantaneous reproductive number, its computation involves the past rather than the future of the dynamics. Therefore, the claim made in Page 4 on the relevance of the future dynamics for this quantity should be modified; nonetheless, both definitions are equivalent as long as the contact and recovery rates remain unchanged (see Nishiura, H., & Chowell, G. (2009). The effective reproduction number as a prelude to statistical estimation of time-dependent epidemic trends. In Mathematical and statistical estimation approaches in epidemiology (pp. 103-121).

We have been more careful in the wording of the description of R – see the second paragraph of section 2.1.

- I like the new part of the discussion at the end of Page 15 on the modifications introduced by Knock et al in the publication following their preprint. Having said this, the description of the interaction between the authors of that paper and the authors of this manuscript are not matter of a scientific work and, therefore, should be removed, leaving possible further subjective interpretations to the reader.

OK, we moved the interaction with Knock et al. to the acknowledgements. If we don't mention it at all then we are open to the criticism 'why didn't you just point all this out to the authors?', especially given the importance of timeliness in getting the science right on this topic.

- Regarding new Figure 6, I would use letters to label each panel to avoid describing them as a function of their position in the figure in the main text.

We have done this (and the same for figure 7).

Reviewer 2

The authors addressed most of my previous comments. In particular, the inclusion of Figure 6 substantially improves the manuscript. Nevertheless, I still have some comments and technical details that are not clear to me.

The intention behind my comments regarding the impact of economic hardship was exactly on what you comment on in the new version of the manuscript. Temporal economic hardship is not the same as endemic/systemic poverty. Therefore, one should not expect the same impact on the health of individuals.

We don't understand what this actually means. We wondered if 'temporary' is meant instead of 'temporal', but even in that case, what are the quantities that are having their impact compared? If 'temporary' is meant we do not understand how it is possible to assert with confidence that the largest shock to the UK economy for 300 years (according to the Bank of England) will result in only temporary hardship. Obviously we hope it is so, but public health policy is about risk management, and should consider the non-negligible risk of things we hope will not happen, such as long term effects.

To give some idea of the scale of the issues, the economic inequality and deprivation related life loss that the current UK population was due to experience, pre-covid, was about 200 million life years (see the data reviewed in Marmot), while the estimates of what may have been saved by the measures amounts to about 3 million life years. Obviously there is much uncertainty, but clearly the economic shock does not have to increase the pre-existing deprivation related losses by a large percentage before the life lost risks exceeding that saved. Note that these life losses are not somehow measuring different things: in both cases (deprivation related of covid) they consist of individuals dying early from ill health (albeit the media attention this generates is wildly different between the cases). One can argue that this is all about political choices and no extra life loss need occur, but since the political choices made in the past have not succeeded in eliminating the 200 million year figure, this seems optimistic, especially when the loss in fact increased after the 2008 shock.

As mentioned in the previous response to referees, we think it is very important to at least *mention* that containment measures carry risks of substantial downsides: this is the reason that it matters that we are careful not to overstate the upsides, if the balance is to be got right. We had hoped not to distract from the main flow of the paper by expanding greatly on this point, but given the instruction to respond to the referee's comment we have expanded the second paragraph of the introduction to point out the evidence in Marmot for life loss consequent on the economic shock of 2008, which suggests that there is at least a risk of similar effects from the current shock. We also state how this compares to government estimates of the life loss that might have been caused by a minimally mitigated Covid epidemic. We discuss life loss in life years, to avoid the many ethical contradictions that arise from instead counting lives lost. This corresponds to the usual practice in the UK when making decisions about allocation of health interventions.

I appreciate the inclusion of the two references on the indirect impact of COVID-19 and the restrictions that were put in place. However, I would like to highlight that neither of the references studies the impact of lockdowns in particular. Both article treat the indirect impacts of COVID-19 in general. The decrease in economic activity cannot be simply attributed to lockdown. The mere existence of a pandemic will affect economic activity, independently on whether restrictions are put in place or not.

Of course. But we think that it is not controversial that, other factors being comparable, the more severe the measures the larger the economic shock. For example, Sweden imposed much lighter measures and experienced a 2020 GDP drop of less than 3% compared to the UK's 10%. Sticking with northern

European countries facing broadly similar situations, German restrictions involved far less closure of economic activity and a GDP drop of 5%. Swiss restrictions were eased (not removed) much more quickly than in the UK and the 2020 GDP drop was again around 3%. (GDP drop underestimates the differences in fact, since it includes the money created through QE as part of economic activity, and this is larger for the UK than for the other countries mentioned).

Furthermore, in the case of England, the closure of restaurants and leisure centres preceded lockdown. I recommend you to make the definition of lockdown more explicit. In particular, point out that the closure of restaurants and nightclubs is not included. As I understand, by lockdown you refer to the closure of non essential retail and the stay-at-home order that was announced on 23 March and took effect on 26 March.

The original version and the current version repeatedly makes the point (e.g. in the Abstract and Discussion) that there were measures in place preceding full lockdown that are likely to have had an effect. These were listed in the Discussion and, in response to a previous referee comment, in the caption of Figure 4, where their timing is first plotted. However, we have now also added the same information to the caption of figure 1, and added the explicit statement that full lockdown involved closure of non-essential services and stay at home orders. Note that although the act formally providing the legal framework for lockdown came into force on the 26th, the government announcement was that the measures applied from 24th, and this is when full lockdown started in practice. Nobody living in the UK would recognise full lockdown as having commenced on the 26 March.

In the reference on South Asia I did not find a part that would justify the conclusion that restrictions led to more deaths than the ones that have been prevented. The only estimation I can find are COVID-19 deaths in 2020 "if no additional mitigation strategies are instituted in the region this year". However, in this case the mitigation measures, including for example the lockdown in India, are already included. Nevertheless, I agree with the authors that the impact of lockdown on health besides COVID-19 should be taken seriously. I just ask the authors to be careful how they communicate this and how studies are interpreted.

The Indian Government, based on work by the public health foundation of India, estimated the lives saved by the Indian lockdown at about 80,000 (PIB India, 2020). For India, the UNICEF report estimates 150,000 extra childhood deaths from the collatoral effects, plus 60,000 still births and 8,000 maternal deaths. Because of the severe increase in risk of death from covid with increasing age and comorbidity load, the average life years lost per covid victim is about 10. Indian life expectancy at birth averages about 70 years, so, even conservatively, a childhood death involves around 5 times the life year loss of a covid death, on average. Despite its low reported covid death rate per capita (0.03%), reported covid deaths in India currently stand a little below 450,000. Obviously the life year loss implied by the UNICEF figures substantially exceeds the approximately 4.5 million life year loss that the direct deaths imply.

Again with some reluctance, we have included these figures in the second paragraph of the introduction. These figures are not and can not be predictions of actual eventual relative losses, but are intended to indicate the scale of the downside risks.

I have a question regarding the incubation period. In section "3.1 - Corrections and minor modifications" the authors state that they use a mean duration of 5.8 days for the incubation period that is equivalent here with the time spent in compartment E. In contrast, in section "3.3 - Relaxing the model assumptions" the authors state that they shorten the E state to have an average of 3 days to infectivity. So which of both the authors actually consider? Or it this the difference between the plot in the top right and bottom left in Figure 6? Also, when the authors shorten the time in E to 3 days do they consider an additional compartment that represents pre-symptomatic infectiousness?

[Note that the model structure is unchanged from the first version of the paper]. We use the literature timings we have quoted. The point is that in reality the exposed, E, stage is **not** the same as the pre-symptomatic stage, despite what Knock et al. (2020) assume. Infected people are infectious before they are symptomatic. To get a generation time consistent with literature estimates you need to allow for this. Our aim was to correct this problem with minimum perturbation of the Knock et al. (2020) model

structure, so we shortened the E stage, set the I stage length to get consistency with the published serial intervals/generation times, and added the P stage to ensure that the average time from infection to hospitalization was consistent with the literature values cited by Knock et al. (2020).

Basically if you want to get inferred timings right, you have to use the correct infection to hospitalisation times, and if you want to get the dynamics right you have to use a plausible latent infection duration and generation time. You can't do both of these if you lock the E duration to the time from infection to symptoms as Knock et al. (2020) do. We suspect that the referees of Knock et al. (2021) may have pointed this out (but that is speculation, we have had no involvement in refereeing any version of the paper, for any journal, and were not approached to do so).

To hopefully help to avoid confusion we have added the following sentence to the end of section 3.3.

> The P state then has an average duration of 5.3 days so that the total time from infection to hospitalization still matches the literature based 5.8 + 6.5 days discussed previously.

The authors comment on the published version of the manuscript by Knock et al. In particular, they mention that in the new version the reproduction number is below one in many regions before lockdown. Looking at their results, it seems that the reproduction number drops below one in many regions on the day the lockdown was announced. I think this is very consistent with the results you have here, even though the day of the announcement is not indicated in the graphics. Obviously, not only the implementation but also the announcement has an impact. This is definitely something you should comment on. I also recommend the authors to include a table where you explicit write when the reproduction number crosses one. It is somehow difficult to see this in the plot but is a crucial result of your analysis. Additionally, I recommend to omit the comments regarding the exchange you had with the authors of Knock et al.. While it may be entertaining academic gossip, I do not think that it adds anything from a scientific perspective. At the end, the community needs to evaluate the content of manuscripts and not their creation process.

Apologies - we had viewed an online version of Knock et al. (2021) where the figure was insufficiently clear and had thought 3 regions showed $R < 1$ prior to lockdown. On detailed inspection of the PDF figure, actually only London does, and we have corrected this.

The announcement of lockdown was made at 20.30 on 23rd of March, so it is hard to see how it can have had a major impact on the 23rd. (The announcement that the prime minister would make an announcement was only made a couple of hours before that, and while locking down was obviously one possibility, it was far from clear in advance what he would say.)

As mentioned in the response to referee 1, we have removed the comments on the exchange with Knock et al from the Discussion, but have mentioned that we provided them with the paper in the Acknowledgements.

Figure 6 now gives the confidence intervals for the point at which R drops below 1 prior to lockdown 1, by region and for the average.

The figure 7 shows the reconstructed incidence. However, I would like to point out that if you assume a non exponential infection model, as it is the case for SARS-CoV-2 (approx. gamma distributed generation time), the reproduction number is not necessarily below one when the peak in infections is reached. As a matter of fact, depending on the decrease, the reproduction number will only drop below one some days afterwards. In this sense, if the peak is reached only a few days before lockdown, the figure you present is not very conclusive. You should comment on the limitation of using exponentially distributed waiting times.

We do not use an exponential distribution for the generation time. There are 3 stages from infection to the end of infectivity, so the generation time will indeed be well approximated by a gamma. This use of sequential stages to generate (exact or approximate) gamma or Erlang distributed lags is standard. As the plots clearly show, our results have the $R = 1$ point after the peak in incidence, as expected.

The peak in the reconstructed incidence in Fig 7a is nine days before lockdown - slightly earlier than our model based estimates, so clearly supporting $R < 1$ before lockdown rather than after. This can

easily be confirmed by applying the method from section 5.1 of Wood (2021) to obtain R reconstructions from the incidence reconstructions. Such a reconstruction is now also shown in figure 7a. This gives $R < 1$ four days before lockdown.

My last comment is regarding the potential change in the evolutionary landscape of the virus that the lockdown may induce. As I understand, your argument applies to almost any measures that try to prevent the spread of the disease. I am thinking about social distancing or contact tracing for example. Accordingly, the conclusion would be to do nothing due to the fear of mutations? Or are there any other possible interventions that may contain the spread where this argument does not apply? I am not experienced in the biology of viruses so I cannot really judge the validity of your argument. However, I do not think it is necessary to motivate your study. I recommend omitting this comment as well as the appendix.

Since this section was included to address comments at the previous round, and neither referee has identified an error in it, we have not followed this suggestion. As mentioned above, we do think that it is important to at least acknowledge what some of the downsides of containment measures may be. We don't understand why this effect would suggest that you should do nothing, when (as we mention) the issue is about the *extent* of suppression of asymptomatic transmission. Even if it did suggest that, why would you then conclude that the effect should not be mentioned? Surely publication of science should not depend on whether or not it supports a particular policy choice? Most risk stratified strategies aimed at mitigation rather that suppression (more akin to Sweden than the UK, for example) will lead to reduced suppression of mixing at low V, less flattening of the curves, and will hence tend to be less favourable to increased V. Stringent self isolation only when symptomatic, but not when asymptomatic, would actually steepen the curve relative to the 'no measures' situation (we now comment on this in the figure caption). On its own the argument in no way determines what strategy should be used - it is simply that the effect should be considered when balancing the risks of a control strategy against its benefits.

References

Knock, E. S., L. K. Whittles, J. A. Lees, P. N. Perez Guzman, R. Verity, R. G. Fitzjohn, K. A. M. Gaythorpe, N. Imai, W. Hinsley, L. C. Okell, A. Rosello, N. Kantas, C. E. Walters, S. Bhatia, O. J. Watson, C. Whittaker, L. Cattarino, A. Boonyasiri, B. A. Djaafara, K. Fraser, H. Fu, H. Wang, X. Xi, C. A. Donnelly, E. Jauneijaite, D. J. Laydon, P. J. White, A. C. Ghani, N. M. Ferguson, A. Cori, and M. Baguelin (2020). Report 41: The 2020 SARS-CoV-2 epidemic in England: key epidemiological drivers and impact of interventions. *Imperial College London*.

Knock, E. S., L. K. Whittles, J. A. Lees, P. N. Perez Guzman, R. Verity, R. G. Fitzjohn, K. A. M. Gaythorpe, N. Imai, W. Hinsley, L. C. Okell, A. Rosello, N. Kantas, C. E. Walters, S. Bhatia, O. J. Watson, C. Whittaker, L. Cattarino, A. Boonyasiri, B. A. Djaafara, K. Fraser, H. Fu, H. Wang, X. Xi, C. A. Donnelly, E. Jauneijaite, D. J. Laydon, P. J. White, A. C. Ghani, N. M. Ferguson, A. Cori, and M. Baguelin (2021). Key epidemiological drivers and impact of interventions in the 2020 SARS-CoV-2 epidemic in England. *Science Translational Medicine*.

PIB India (2020, May). Government of India Press Briefing on the actions taken, preparedness and updates on COVID-19, 22nd May 2020, Press Information Bureau. `https://pib.gov.in/WebcastMore.aspx?webcast_tempID=434`.

Wood, S. N. (2021). Inferring UK COVID-19 fatal infection trajectories from daily mortality data: were infections already in decline before the UK lockdowns? *Biometrics*.
